# Leaf traits in Chilean matorral: sclerophylly within, among, and beyond matorral, and its environmental determinants

**DOI:** 10.1002/ece3.1970

**Published:** 2016-02-03

**Authors:** Jennifer Read, Gordon Sanson, María Fernanda Pérez Trautmann

**Affiliations:** ^1^ School of Biological SciencesMonash UniversityMelbourneVictoria3800Australia; ^2^ Departamento de EcologíaFacultad de Ciencias BiológicasPontificia Universidad Católica de ChileSantiagoChile

**Keywords:** Chile, ET_0_, leaf toughness, leaf traits, matorral, sclerophylly, soil nutrients, soil phosphorus

## Abstract

Studies of leaf traits often focus on tradeoffs between growth and resource conservation, but little is known about variation in the mechanical traits that influence resource conservation. This study investigates how leaf mechanical traits vary across matorral vegetation in central Chile, how they correlate with environmental factors, and how these trends compare at a broader geographic scale. Leaf toughness, strength, stiffness, and associated traits were measured in five matorral types in central Chile, and relationships with soil N and P and climate variables were assessed. Trends with soil and climate were then analyzed across shrubland and woodland in Chile, Western Australia, and New Caledonia. Chilean species varied in leaf mechanics and associated traits, both within and among matorral types, with more species in sclerophyll matorral having strong, tough, and stiff leaves than in arid and littoral matorral. Overall, leaves with high leaf dry mass per area were stiffer, tougher, stronger, thicker, denser, with more fiber, lignin, phenolics and fiber per unit protein and less protein: tannin activity and N and P per mass, forming a broad sclerophylly syndrome. Mechanical traits of matorral species were not correlated with soil N or P, or predictably with climate variables, except flexural stiffness (*EI*_W_) which correlated positively with annual reference evapotranspiration (ET_0_). However, soil P made strong independent contributions to variation in leaf mechanics across shrublands and woodlands of Chile, Western Australia, and New Caledonia, either separately (strength) or together with ET_0_ (toughness) explaining 46–90% of variation. Hence ET_0_ was predictive of *EI*_W_ in Chilean matorral, whereas soil P was highly predictive of variation in leaf strength, and combined with ET_0_ was highly predictive of toughness, at a broader geographic scale. The biological basis of these relationships, however, may be complex.

## Introduction

Both climate and soil nutrition appear to be strong selective forces on leaf texture, reflecting tradeoffs between growth and resource conservation (Wright et al. 2005; Ordoñez et al. 2009). Sclerophylly, a leaf textural form (hard, tough, stiff, leathery leaves: Schimper 1903) at the slow‐return end of the suggested leaf economics spectrum (Wright et al. 2005), is common in mediterranean climates (Schimper 1903; Turner 1994). In particular, sclerophylly has been suggested to be an adaptation to seasonal drought, with, for example, stiff cell walls facilitating turgor maintenance (Oertli et al. 1990), and sclerenchyma bundle sheath extensions and thick cuticle and leaves contributing to water conservation (Heide‐Jørgensen 1990). Arguably, under the drought resistance hypothesis, sclerophylly (a leaf‐level textural trait) is not adaptive *per se* but rather a consequence of anatomical traits that are adaptations to seasonal drought.

Sclerophylly is also common in vegetation on low‐nutrient soils across a range of climates, including moist tropical (Loveless 1961; Sobrado and Medina 1980; Grubb 1986; Specht and Rundel 1990; Choong et al. 1992; Turner 1994; Lamont et al. 2002). Some anatomical features may enhance nutrient conservation on low‐nutrient soils, for example, reduced leaching through a thick cuticle, but sclerophylly may provide direct benefits by conferring protection, particularly against herbivores, since enhanced LLS (leaf life span) is advantageous in conditions where it takes longer to maximize returns on investment (Turner 1994; Westoby et al. 2002). Indeed, protection from herbivores and consequent resource conservation may be cost‐effective in a wide range of stressful environments (Turner 1994). A fourth alternative is that sclerophylly is just a consequence of constraints imposed by severe environments, for example, more limited synthesis of protein than carbohydrate on low‐nutrient soils (Salleo and Nardini 2000). Most probably, sclerophylly is a complex and variable syndrome of traits, to which all these factors may contribute.

Leaf trait studies usually focus on the growth–resource conservation tradeoff (Díaz et al. 2004; Wright et al. 2005; Ordoñez et al. 2009), with less emphasis on specific mechanical traits that contribute to resource conservation (but see Choong et al. 1992; Read et al. 2005, 2006; Onoda et al. 2011; Méndez‐Alonzo et al. 2013). Furthermore, there are few datasets that allow detailed comparison of the mechanical traits that contribute to sclerophylly, and how they differ in contrasting environments, and indeed of any leaf economic traits in relation to soil variables across large spatial scales (Ordoñez et al. 2009; Maire et al. 2015). Comparative studies of mechanical traits are of value in resolving the relative importance of adaptive and “nonadaptive” traits and hence better understand the forces leading to the prevalence of sclerophylly in certain environments and the consequences for growth–resource conservation tradeoffs. In particular, it is not clear how sclerophylly varies in different environments, that is, whether leaves have similar mechanics, morphology, anatomy, and chemistry, relate to environmental conditions in similar ways (e.g., Wright and Westoby 2002; Méndez‐Alonzo et al. 2013), and have similar influences on, for example, plant–animal interactions, nutrient cycling, and carbon economy (Díaz et al. 2004; Peeters et al. 2007; Méndez‐Alonzo et al. 2013).

The matorral vegetation of Chile provides a useful contribution to this discussion since distinct forms of vegetation occur across a range of mediterranean‐type climates and soils, including, but not limited to, sclerophyll communities (Rundel 1981; Armesto et al. 2007). Furthermore, it contrasts with sclerophyll vegetation in some regions by having more fertile soil (Lamont 1994), so is predicted by the nutrient conservation hypothesis to have softer leaves than, for example, the heath vegetation of Western Australia. Here we address the general hypothesis that leaf mechanical traits such as strength, toughness, and stiffness increase along gradients of water deficit and/or soil nutrient deficiency. We investigate (1) how leaf toughness, strength, and stiffness, and associated traits, vary among differing matorral vegetation of central Chile,and (2) the potential influence of soil nutrients and climate factors on leaf trait syndromes. We then compare these data with those recorded in sclerophyll vegetation in mediterranean climates of southwest Australia and tropical climates of New Caledonia, over a range of soil fertility, testing (3) the relative contribution of soil nutrients versus climate variables to variation in mechanical traits at this broader geographic scale.

## Materials and Methods

### Study region

Mediterranean‐type vegetation, or matorral, occurs in central Chile at 30–36°S (Armesto et al. 2007). Evergreen sclerophyll shrubland is the most common form, particularly on the slopes of the Coastal Cordillera and the lower slopes of the Andes, but matorral varies considerably, occurring across varying climates and soils (Armesto et al. 2007). Mean annual maximum temperatures for the matorral zone are *c*. 20–25°C, with extremes moderated near the coast (Rundel 1981). Annual rainfall is *<*200–700 mm, mostly during the coolest months (April–September), with a long dry spring–summer of up to 6 months (Armesto et al. 2007). In some regions, fog provides an important source of moisture, particularly in summer (Armesto et al. 2007; Negret et al. 2013).

### Study sites

**Figure 1:**
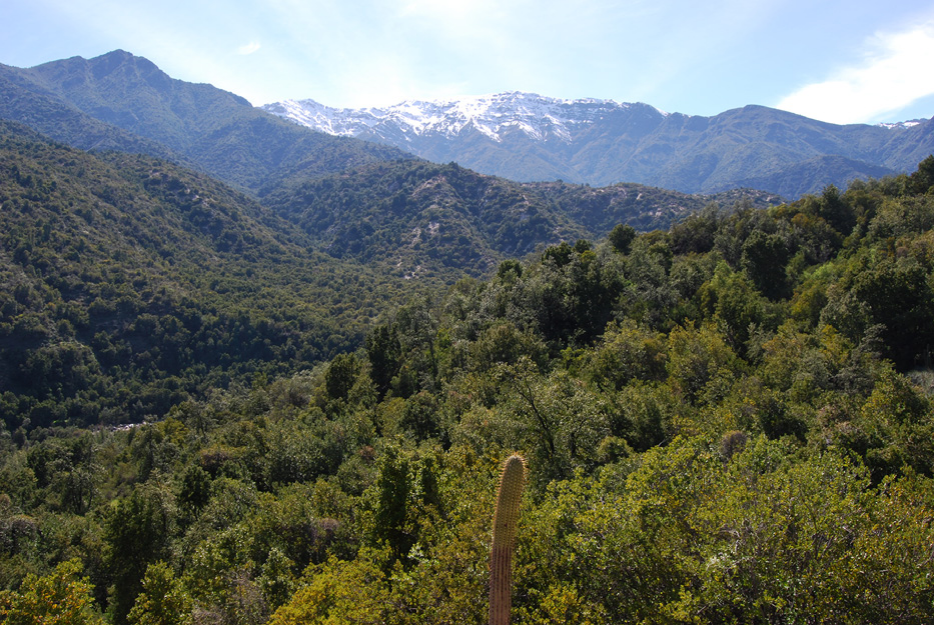
Mid‐elevation matorral at Reserva Nacional Río Clarillo, south‐central Chile.

Five types of matorral were selected, following the main types described by Rundel (1981), across contrasting environments from the coast to the lower mountains of the Andes at *c*. 31–34°S (Table 1, Appendix). They were sampled in September (austral spring) 2008, including only woody dicots and subshrubs and excluding succulents. Littoral matorral was sampled on coastal bluffs at two sites near Zapallar in shrubland dominated by malacophyllous shrubs, including summer‐deciduous species, interspersed with *Puya* spp. (Bromeliaceae) and cacti. Lowland sclerophyll matorral was sampled at two sites *c*. 2 km from the sea at Cachagua, dominated by evergreen sclerophyll shrubs of *c*. 1–5 m high with small trees to *c*. 10 m high. Both the Zapallar and Cachagua sites commonly experience morning fog. Mid‐elevation sclerophyll matorral was studied at Reserva Nacional Río Clarillo in open sclerophyll shrubland, including vegetation along drainage lines (Fig. 1). Montane sclerophyll matorral of low shrubs with infrequent tall shrubs/small trees was sampled at Santuario de la Naturaleza Yerba Loca. Arid matorral of short shrubs with sparse small trees and frequent cacti was studied at Reserva Nacional Las Chinchillas.

**Table 1:** Environmental comparisons of the Chilean matorral vegetation types. The soil data are means ± standard errors. See text for sources of climate data. Results of ANOVA are presented, based on log‐transformed data

	Arid matorral	Littoral matorral	Lowland sclerophyll matorral	Mid‐elevation sclerophyll matorral	Montane sclerophyll matorral
Study area	Las Chinchillas	Zapallar	Cachagua	Río Clarillo	Yerba Loca
Lat/long	31˚30.7′S, 71˚6.4′W	32˚33.1′S 71˚28.1′W	32˚35.6′S, 71˚25.1′W	33˚43.7′S, 70˚ 28.4′W	33˚20.3′S, 70˚20.1′W
Distance to sea (km)	44	0	2	108	120
Elevation (m asl)	550–700	5–30	100–180	900–950	1700–1780
AMT (°C)	14.3	15.7	15.7	14.2	12.7
AP (mm)	212	357	357	497	445 (at 2500 m asl)
PDQ (mm)	2	4	2	13	20
ET_0_ (mm)	1103	926	921	1118	1245
*Soil properties*					
pH**	7.8 ± 0.3^a^	7.2 ± 0.4^ab^	6.3 ± 0.3^b^	6.3 ± 0.1^b^	7.3 ± 0.1^ab^
LOI (%)***	4.2 ± 0.4^a^	4.7 ± 0.4^a^	8.5 ± 0.8^b^	4.1 ± 0.7^a^	9.6 ± 0.4^b^
Nitrogen (mg g^−1^)**	0.61 ± 0.09^ab^	0.83 ± 0.15^ab^	1.37 ± 0.26^a^	0.34 ± 0.13^b^	0.86 ± 0.04^ab^
Phosphorus (mg g^−1^)***	0.74 ± 0.06^a^	0.54 ± 0.05^a^	0.62 ± 0.09^a^	0.20 ± 0.02^b^	0.67 ± 0.06^a^

AMT, annual mean temperature; AP, annual precipitation; PDQ, precipitation of the driest quarter; ET_0_, annual reference evapotranspiration; LOI, loss on ignition, an estimate of soil organic content.

Asterisks indicate *P*‐values: **P <* *0.01; ***P <* *0.001. Shared alphabet letters indicate no significant difference. Latitudes and longitudes are given for a single collection area within the study site. Precipitation variables for Zapallar and Cachagua do not take supplementary humidity due to fog into account.

Climate data (annual precipitation and annual mean temperature) were obtained for study areas from local meteorological stations, with monthly precipitation estimated by WorldClim v. 1.4 (Hijmans et al. 2005; 30 arc seconds resolution) with DIVA‐GIS v. 7 (Hijmans et al. 2012). Penman–Monteith reference evapotranspiration rate (ET_0_) was estimated by the 10‐arcmin IWMI World Water Climate Atlas (http://www.iwmi.cgiar.org/). Soil samples were collected at three to five semi‐randomly located points in each matorral. Each sample comprised two to five subsamples, taken at least 5 m apart, from the top 10 cm of soil, and was air‐dried and sieved to 2 mm. Soil organic concentration was measured by LOI (loss on ignition) of a sample combusted at 550°C for 2 h. Soil pH was measured using a 1:5 mass: volume mixture. Total N and P were measured as for leaves (see below), on samples ground to 0.2 mm.

### Sample collection

Each matorral type was sampled at five locations across at least 1 km, sampling each species at each location when possible. Healthy, mature sunlit leaves were collected from up to five plants of the 10–25 most common shrubs and trees. A total of 62 species was collected, with 13 sampled in more than one type of matorral. We aimed to sample leaves that were *c*. 6–12 months old, produced during the last growing season, and having experienced winter. However, leaves of summer‐deciduous species were *c*. 3 months old, produced during winter (e.g., Aljaro et al. 1972), and the oldest leaves of some subshrubs were possibly also produced during winter. Hence, although all leaves were mature, some differed in age and exposure to seasons. Mechanical and morphological traits and water content were measured on fully hydrated leaves within 12–48 h of collection. Leaves used for chemical analyses were air‐dried, then oven‐dried at 40°C. Potential LLS (age to senescence) was estimated in three shoots per plant, based on twig morphology and color, bud scars, and senescing leaves.

### Leaf morphological and mechanical traits

One to five leaves per plant were weighed fresh, without petioles. Lamina thickness was measured at three random locations, avoiding the midrib, with a micrometer or calipers depending on leaf morphology. Leaf area was measured by image analysis (Mix Image analysis v. 3, Monash University, Victoria, Australia). Leaves were then oven‐dried at 40°C and reweighed to calculate water content, LMA (leaf dry mass per area) which is a commonly used index of sclerophylly (Groom and Lamont 1999), and tissue density.

Relatively little is known about how mechanical traits derived from differing test types (punch, shear, tear and bending) vary within and among vegetation types, including in sclerophyll vegetation. Hence, we investigated multiple leaf traits, derived from the main test types (e.g., Read and Sanson 2003). Leaf strength, toughness, and stiffness were measured following Read and Sanson (2003), using a portable custom‐made force tester (Read et al. 2005, 2006). Strength was measured as maximum force to fracture using a punch test (0.5 mm diameter, sharp‐sided punch), with five random punches across the lamina, avoiding the midrib. Toughness was measured as work to fracture, calculated as the area under the force–displacement curve generated by punch, shear, and tear tests. Shear tests (20° blade approach angle) were undertaken on a strip cut from one side of the leaf, avoiding the midrib and margins. The flexural Young's modulus (*E,* a “material property”) and flexural stiffness (*EI*, a “structural property” that includes the effect of leaf thickness, where *I* is the second moment of area) were measured on a leaf strip using a three‐point bending test with a span:depth ratio of 70. *EI*_W_ was calculated as flexural stiffness per unit strip width. Tear strength and toughness were measured on notched leaf strips with an aspect ratio >10 (Vincent 1990), mounted in clamps, and secured with cyanoacrylate glue. Results for fracture tests were also expressed per unit leaf thickness (“material properties”), termed “specific work” and “specific strength” (Read and Sanson 2003). Leaf thickness was measured at the point of fracture, with a micrometer or calipers. Tearing and bending tests could not be undertaken in some small‐leaved species (Appendix).

### Leaf chemistry

Leaves were freeze‐dried, then ground to a powder. Foliar N was measured by a Leco CHN‐200 auto‐analyser (Leco Corp., St Joseph, MI) and P by the molybdenum‐blue colorimetric method (Grimshaw et al. 1989) after digestion by the sulfuric‐peroxide procedure (Grimshaw 1987). N and P were expressed per unit dry mass (N_mass_, P_mass_) and per unit mass of water (N_water_, P_water_), the latter allowing an estimate of protoplasmic concentration. “Total phenolics” were extracted in acetone (Cork and Krockenberger 1991) and assayed by the Prussian blue method (Price and Butler 1977) with concentration expressed as GAE (gallic acid equivalents) per leaf dry mass. Tannin activity was estimated as the amount of bovine serum albumen bound by the phenolic extract (Asquith and Butler 1985), expressed as mass of protein precipitated per unit leaf dry mass. Total cell wall, measured as NDF (neutral detergent fiber), and ADL (acid detergent lignin) were quantified following Van Soest et al. (1991). The Loveless sclerophylly index (SI) was calculated as NDF per unit protein (N_mass_ × 6.25).

### Data analysis

Differences among sites were tested by ANOVA with Tukey's *post hoc* comparisons. Pearson correlation was used to test associations among leaf traits, averaging values of species sampled in multiple vegetation types. Log transformations were used when necessary to improve data normality, reduce the influence of outliers, and improve linearity for correlations and regressions. PCA (Principal components analysis) was used to summarize data (species' averages) into main components, first using the full data set of species averages for each site, then averaging those species sampled in multiple vegetation types. Only traits measured in all species were included, that is, excluding traits from tearing and bending tests. Since there was little difference in results, PCA of the full data set is presented to show within‐species variation across sites. Spearman correlation was used to test associations of leaf traits with environmental variables.

Comparisons were made with mediterranean‐climate shrubland and woodland in southwest Western Australia (Read et al. 2005) and tropical moist shrubland on ultramafic soils and dry forest (excluding lianes) in New Caledonia (Read et al. 2006), these studies having used identical methods to measure leaf mechanics and providing contrasts in climate and soil fertility. For example, shrubland study sites in southwest Western Australia and New Caledonia have lower soil phosphorus concentrations (<0.2 mg g^−1^) than in Chilean matorral (≥0.2 mg g^−1^), and the New Caledonian sites have higher annual rainfall (>900 mm) than the Australian and Chilean sites (<500 mm).

ANCOVA was used to test the relationship of work to punch with LMA among regions, across an LMA range of 100–300 g m^−2^ (where there was most overlap among regions). Trait variation in evergreen species was summarized across regions with PCA, excluding the littoral matorral in Chile because of likely influences of salinity and wind exposure. Pearson correlation was used to test associations of selected leaf traits with environmental variables across regions. Of the mechanical traits, only punch tests were included, since shear tests could not always avoid the midrib in small‐leaved species, so were less comparable across species, and stiffness and tear tests could not be undertaken in small‐leaved species. Hierarchical partitioning (Chevan and Sutherland 1991) was used to calculate independent contributions (*I*_HP_) of environmental variables to explained variance in mechanical traits and LMA, with statistical significance determined by randomization techniques (Walsh and Mac Nally 2013). Linear regression was then used to determine the variance explained individually or together of significant variables. Hierarchical partitioning was undertaken with hier.part (Walsh and Mac Nally 2013) in R 2.14.1 (R Development Core Team, 2011). SYSTAT v. 13 (Systat Software Inc., San Jose, CA) was used for other analyses, with *α *= 0.05 for hypothesis testing.

## Results

### Soil nutrients

Soils were generally neutral or weakly acidic, with low organic content, and low levels of P and particularly N (Table 1). There were some differences among vegetation types, notably in higher LOI at the lowland and montane sclerophyll matorral, and low N and P at mid‐elevation sclerophyll matorral (Table 1).

### Plant and leaf traits across species and sites

Deciduous and semi‐deciduous species occurred in all types of matorral, but more were recorded in arid matorral (Table 2). Some evergreen species, particularly subshrubs, had leaves only on soft young stems, suggesting LLS of <6 months. Overall, 1+ years (≥1 year, but <2 years) (43% of species) was the modal LLS, with 38% of species having LLS <1 year, and only 20% of species having LLS of 2–3 years. A high proportion of species in littoral matorral had short‐lived leaves, with fewest in mid‐elevation sclerophyll matorral (Table 2).

**Table 2:** Comparisons of leaf traits among the Chilean matorral vegetation types. The data are means of species' means ± standard errors. The results of ANOVA are given, with *post hoc* Tukey's tests (shared alphabet letters indicate no significant difference among sites)

Trait	Arid matorral	Littoral matorral	Lowland sclerophyll matorral	Mid‐elevation sclerophyll matorral	Montane sclerophyll matorral	*F*	*P*
Deciduous (% of species)	40	20	8	9	10		
Leaf life span (% <1 year)	50	78	40	19	34		
*Leaf chemistry and morphology*							
N_mass_ (%) _L_	2.48 ± 0.23^a^	2.35 ± 0.21^a^	1.80 ± 0.20^a^	1.75 ± 0.23^a^	2.01 ± 0.35^a^	2.6	**0.045**
N_water_ (mg g^−1^ water) _L_	11.51 ± 0.60^ac^	4.31 ± 0.30^b^	9.28 ± 0.67^c^	11.80 ± 0.69^a^	14.80 ± 1.21^a^	31.5	**<0.001**
P_mass_ (mg g^−1^) _L_	2.10 ± 0.25^ab^	3.25 ± 0.41^b^	1.84 ± 0.19^a^	1.39 ± 0.15^a^	1.85 ± 0.23^a^	6.4	**<0.001**
P_water_ (mg g^−1^ water) _L_	1.02 ± 0.12^ac^	0.61 ± 0.09^b^	0.97 ± 0.09^c^	0.98 ± 0.06^ac^	1.47 ± 0.22^a^	6.8	**<0.001**
Nitrogen: phosphorus	13.8 ± 2.0^a^	8.4 ± 0.8^b^	10.9 ± 0.7^ab^	13.1 ± 0.6^a^	12.7 ± 1.6^ab^	3.7	**0.009**
Carbon: nitrogen	19.7 ± 3.0^a^	19.7 ± 2.0^a^	29.9 ± 3.2^ab^	34.0 ± 3.1^b^	27.6 ± 3.2^ab^	3.3	**0.016**
NDF (%) _L_	21.4 ± 2.9	20.7 ± 1.7	25.4 ± 2.0	24.1 ± 2.1	20.4 ± 2.1	0.8	0.553
ADL (%) _L_	4.8 ± 0.8	4.1 ± 0.6	8.2 ± 1.4	8.0 ± 1.2	7.8 ± 1.3	2.1	0.089
Total phenolics (g GAE. 100 g^−1^) _L_	2.9 ± 0.9	2.6 ± 0.8	4.4 ± 0.6	4.6 ± 0.6	4.6 ± 1.0	2.0	0.105
Protein precipitation (mg g^−1^) _L_	145 ± 113^ab^	76 ± 55^b^	223 ± 51^a^	241 ± 53^a^	229 ± 74^a^	4.4	**0.003**
Protein: protein precipitation (g g^−1^) _L_	6.6 ± 1.5	6.6 ± 1.3	3.3 ± 0.9	3.1 ± 0.8	3.0 ± 1.3	2.3	0.063
Water (g g^−1^) _L_	2.19 ± 0.17^a^	5.58 ± 0.46^b^	2.18 ± 0.31^a^	1.46 ± 0.14^a^	1.39 ± 0.23^a^	18.6	**<0.001**
Leaf size (mm^2^) _L_	308 ± 99^a^	729 ± 194^ab^	1163 ± 201^b^	959 ± 197^b^	481 ± 99^ab^	4.6	**0.002**
Thickness (mm) _L_	0.36 ± 0.08	0.35 ± 0.06	0.29 ± 0.02	0.34 ± 0.03	0.41 ± 0.04	1.5	0.222
Tissue density (mg mm^−3^) _L_	0.350 ± 0.027^a^	0.206 ± 0.017^c^	0.421 ± 0.025^ab^	0.473 ± 0.022^b^	0.480 ± 0.028^b^	14.6	**<0.001**
LMA (g m^−2^) _L_	124 ± 29^ab^	63 ± 8^b^	127 ± 14^a^	159 ± 14^a^	196 ± 22^a^	7.0	**<0.001**
SI (g g^−1^) _L_	1.52 ± 0.24	1.69 ± 0.24	3.29 ± 0.53	3.15 ± 0.47	2.16 ± 0.37	1.5	0.200
*Leaf mechanics*							
Work to shear (J m^−1^) _L_	0.144 ± 0.023	0.106 ± 0.022	0.151 ± 0.020	0.200 ± 0.045	0.173 ± 0.035	0.8	0.524
Specific work to shear (kJ m^−2^) _L_	0.456 ± 0.078	0.343 ± 0.033	0.498 ± 0.050	0.576 ± 0.107	0.419 ± 0.078	0.8	0.512
Punch strength (MN m^−2^) _L_	4.43 ± 0.56^ab^	2.94 ± 0.38^a^	5.86 ± 0.75^ab^	6.67 ± 0.93^b^	5.34 ± 0.67^ab^	3.0	**0.025**
Specific punch strength (GN m^−2^ m^−1^) _L_	18.6 ± 4.2^ab^	12.5 ± 1.5^a^	23.3 ± 2.2^b^	22.6 ± 2.6^b^	14.9 ± 1.6^ab^	4.3	**0.004**
Work to punch (kJ m^−2^) _L_	1.22 ± 0.25	0.93 ± 0.19	1.27 ± 0.18	1.60 ± 0.27	1.63 ± 0.24	1.5	0.202
Specific work to punch (MJ m^−2^ m^−1^) _L_	4.23 ± 0.76	3.44 ± 0.32	4.79 ± 0.46	5.10 ± 0.66	4.41 ± 0.57	1.0	0.418
Tear strength (MN m^−2^) _L_	1.46 ± 0.62	0.68 ± 0.10	1.44 ± 0.16	1.52 ± 0.31	1.03 ± 0.17	2.2	0.078
Work to tear (J m^−1^) _L_	0.349 ± 0.018	0.193 ± 0.063	0.244 ± 0.041	0.303 ± 0.054	0.240 ± 0.053	0.7	0.580
Specific work to tear (kJ m^−2^) _L_	1.25 ± 0.34	0.58 ± 0.11	0.82 ± 0.11	0.78 ± 0.11	0.57 ± 0.14	1.4	0.264
*E* (MN m^−2^) _L_	177 ± 60	75 ± 6	232 ± 37	241 ± 58	164 ± 42	1.7	0.144
*EI*_W_ (mN m^2^ m^−1^) _L_	1.01 ± 0.65^ab^	0.17 ± 0.05^b^	1.06 ± 0.34^ab^	1.82 ± 0.69^ab^	2.01 ± 0.58^a^	2.8	**0.035**

L, log‐transformed for analysis. NDF, neutral detergent fiber; ADL, acid detergent lignin; GAE, gallic acid equivalents; LMA, leaf dry mass per area; SI, Loveless sclerophylly index; *E*, Young's modulus; *EI*_W_, flexural stiffness.

Significant *P*‐values are shown in bold.

#### Foliar nutrients, cell wall and phenolics

Foliar N_mass_ and P_mass_ varied six‐ to ninefold among species, with a trend of lowest concentrations in sclerophyll matorral (Table 2). N_water_ and P_water_ differed among vegetation types, with low values in littoral matorral (Table 2). N:P ratios varied fourfold among species, with one‐third having N:P ratios <10 (particularly in littoral matorral), suggesting likely N limitation (Güsewell 2004). There was little clear evidence of P limitation (N:P > 20: Güsewell 2004), with the highest N:P of 20–21 recorded in only four species. C:N ranged from 10 in some deciduous species to *c*. 60 in numerous evergreen species, with higher values in mid‐elevation sclerophyll matorral than in arid and littoral matorral (Table 2).

Neutral detergent fiber varied *c*. fivefold among species, but did not differ among vegetation types (Table 2). ADL varied 10‐fold among species, with no vegetation differences, but the trend was for higher values in sclerophyll matorral (Table 2). Total phenolics varied 16‐fold among species, with no differences among vegetation types, although the trend was also for higher values in sclerophyll matorral (Table 2). Tannin activity (protein precipitation), with very high variability among species (>300‐fold), was higher in sclerophyll matorral than littoral matorral (Table 2). The ratio of protein: tannin activity, an estimate of protein availability to herbivores, also varied 300‐fold among species, with a trend of lowest values in sclerophyll matorral (Table 2).

#### Leaf morphology, sclerophylly indices and mechanics

Water content varied 12‐fold among species, highest in littoral matorral (Table 2). Leaf size varied from <20 mm^2^ to >4000 mm^2^, with smaller leaves in arid matorral than lowland and mid‐elevation sclerophyll matorral (Table 2). LMA varied from 24 g m^−2^ to >300 g m^−2^, lower in littoral matorral than in sclerophyll matorral (Table 2). Of its components, tissue density in littoral matorral was less than half that of sclerophyll matorral, but leaf thickness did not differ among vegetation types (Table 2). SI varied *c*. 20‐fold among species, but not among vegetation types, although the trend was for highest values in sclerophyll matorral (Table 2).

**Figure 2:**
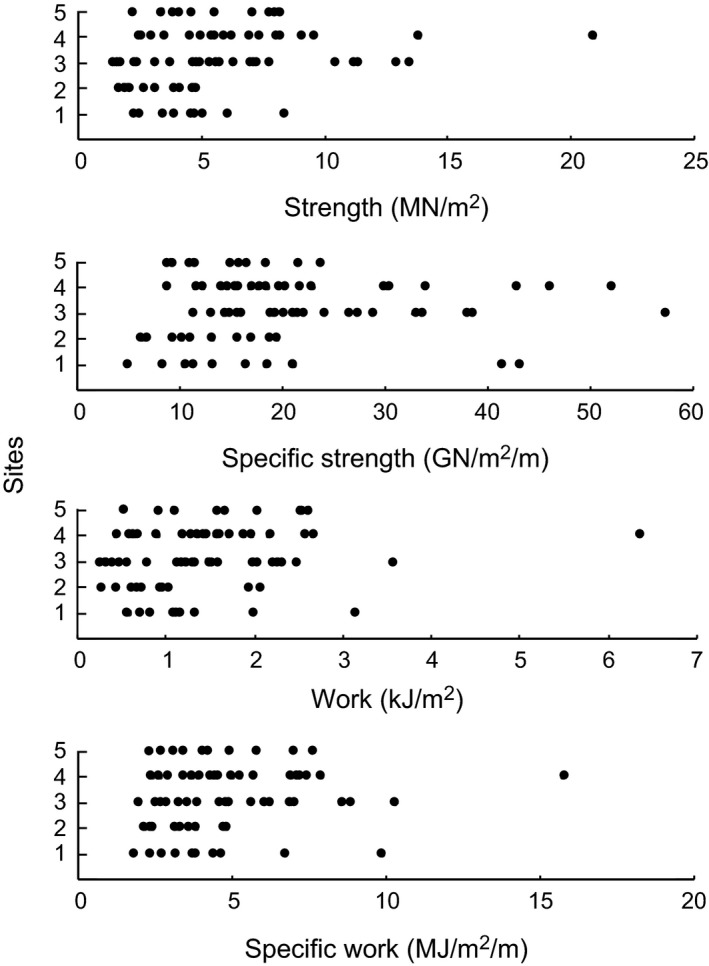
Comparison of mechanical variables from punch tests among matorral types in Chile. Dots represent average values of individual species. (1) Arid matorral; (2) littoral matorral; (3) lowland scler ophyll matorral; (4) mid‐elevation sclerophyll matorral; (5) montane sclerophyll matorral.

There was little significant difference in mechanical traits among vegetation types (Table 2), although there was 16‐ to 950‐fold variation among species. Punch strength varied 41‐fold among species and was *c*. twofold higher in mid‐elevation sclerophyll matorral than littoral matorral, with a trend for higher values in sclerophyll matorral (Table 2). Specific punch strength ranged 24‐fold, higher in lowland and mid‐elevation sclerophyll matorral than in littoral matorral (Table 2). Flexural stiffness, *EI*_W_, was higher in montane sclerophyll matorral than in littoral matorral (Table 2) and varied 950‐fold among species. No other mechanical traits differed among vegetation types, despite 20‐ to 70‐fold variation among species (Table 2). However, examination of mechanical data showed wide variation within matorral types, particularly in coastal and mid‐elevation sclerophyll matorral, but with sclerophyll matorral typically showing a greater proportion of high values of each mechanical property than arid and littoral matorral (Fig. 2 for punch variables). Relatively few differences in conclusions were reached when deciduous and semi‐deciduous species were excluded from analyses, and these were generally minor (Appendix S1).

Leaf traits differed in magnitude among LLS classes, with the exception of N_water_ and P_water_ (Table 3). Overall, short‐lived leaves had higher N_mass_ and P_mass_, high protein: tannin activity, and lower NDF, ADL, phenolics, tannin activity, LMA, strength, specific strength, toughness, specific toughness, and stiffness (Table 3). Thus, they are potentially more nutritious and less defended, at least by these carbon‐based defenses, than longer‐lived leaves.

**Table 3:** Variation in selected leaf traits among leaf life span classes of Chilean matorral species. Potential leaf life spans of each species (across all types of matorral) were categorized as <1 year, 1+ years (≥1 year but <2 years) and 2–3 years. The values presented are means of species' means ± standard errors. All data were log‐transformed (_L_) for analysis

Leaf trait	Leaf life span (years)			*F*	*P*
	<1	1+	2–3		
N_mass_ (%) _L_	2.79 ± 0.19^a^	1.56 ± 0.09^b^	1.00 ± 0.05^c^	54.0	**<0.001**
N_water_ (mg g^−1^ water) _L_	10.5 ± 1.2	10.7 ± 0.7	10.6 ± 0.6	0.5	0.578
P_mass_ (mg g^−1^) _L_	2.72 ± 0.11^a^	1.51 ± 0.13^b^	0.98 ± 0.06^c^	43.3	**<0.001**
P_water_ (mg g^−1^ water) _L_	1.00 ± 0.10	1.04 ± 0.11	1.05 ± 0.07	0.3	0.757
NDF (%) _L_	18.4 ± 1.5^a^	23.0 ± 1.3^b^	34.6 ± 0.3^c^	14.6	**<0.001**
ADL (%) _L_	4.8 ± 0.9^a^	8.3 ± 1.2^b^	8.7 ± 1.4^b^	7.5	**<0.001**
Total phenolics (g GAE. 100 g^−1^) _L_	2.4 ± 0.3^a^	4.8 ± 0.6^b^	5.7 ± 0.6^b^	12.2	**<0.001**
Protein precipitation (mg g^−1^) _L_	69 ± 22^a^	252 ± 53^b^	339 ± 65^b^	9.2	**<0.001**
Protein: protein precipitation (g g^−1^) _L_	6.4 ± 0.8^a^	3.0 ± 0.7^b^	0.6 ± 0.2^c^	17.5	**<0.001**
LMA (g m^−2^) _L_	79 ± 9^a^	168 ± 13^b^	209 ± 18^b^	31.7	**<0.001**
Punch strength (MN m^−2^) _L_	3.0 ± 1.3^a^	5.9 ± 1.2^b^	14.1 ± 1.7^c^	42.3	**<0.001**
Specific punch strength (GN m^−2^ m^−1^) _L_	15 ± 3^a^	19 ± 3^a^	39 ± 4^b^	10.8	**<0.001**
Work to punch (kJ m^−2^) _L_	0.7 ± 0.4^a^	1.6 ± 0.4^b^	3.6 ± 0.6^c^	29.9	**<0.001**
Specific work to punch (MJ m^−2^ m^−1^) _L_	3.4 ± 0.8^a^	4.9 ± 0.8^b^	9.5 ± 1.1^c^	15.5	**<0.001**
*EI*_W_ (mN m^2^ m^−1^) _L_	0.3 ± 0.4^a^	1.3 ± 0.4^b^	3.7 ± 0.6^c^	20.5	**<0.001**

NDF, neutral detergent fiber; ADL, acid detergent lignin; GAE, gallic acid equivalents; LMA, leaf dry mass per area; *EI*_W_, flexural stiffness.

Results are given for ANOVA (significant *P*‐values are shown in bold) with *post hoc* Tukey's tests where appropriate (shared alphabet letters indicate no significant difference). Note that the leaves in the two upper size classes were the same age at the time of sampling, but that leaves in the shorter life span class were younger (<1 year old) although mature at the time of sampling.

### Correlations among leaf traits and with environmental variables across species

**Figure 3:**
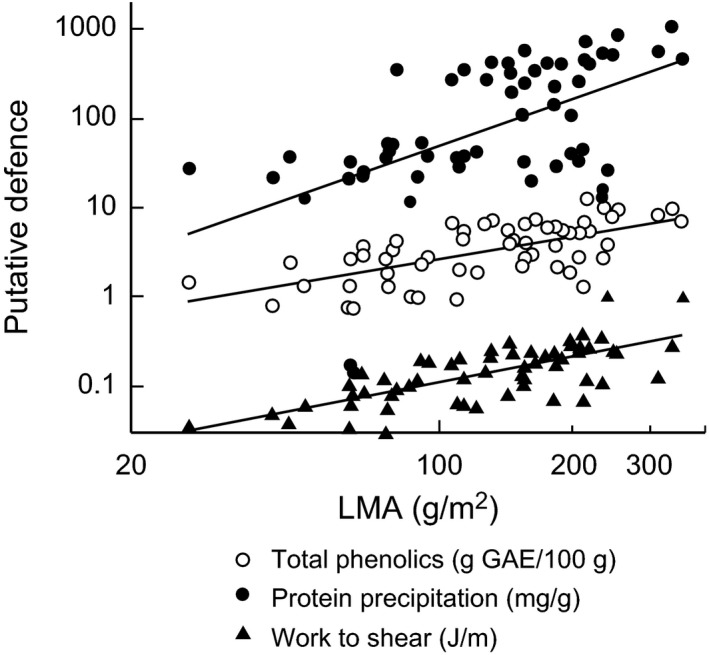
The relationship between leaf dry mass per area and putative defenses (total phenolics, tannin activity (protein precipitation), and work to shear) of evergreen species across matorral types in Chile. Work to shear was chosen from the suite of measured mechanical traits to allow comparison with relationships measured by Read et al. (2009). Species sampled at multiple vegetation types are included as a single data point (averaged values). GAE, gallic acid equivalents.

All mechanical traits were strongly positively intercorrelated (*P *≤* *0.001), except for a weak correlation of *EI*_W_ with specific work to tear (*r *=* *0.30, *P *=* *0.034) and no correlation with specific punch strength (*r *=* *0.22, *P *=* *0.125) (Appendix S2). For simplicity, tearing variables and *E* will not be considered further. The sclerophylly indices, LMA and SI, were positively correlated (*r *=* *0.67, *P *<* *0.001). All mechanical traits except specific punch strength were strongly positively correlated with LMA, particularly *EI*_W_, and with SI (Table 4). All nonmechanical traits except N:P and leaf size correlated with LMA (Table 4). Notably, while N_mass_ and P_mass_ correlated strongly and negatively with LMA (*r *=* *−0.78, −0.68, respectively; *P *<* *0.001), N_water_ and P_water_ correlated less strongly and positively with LMA (*r *=* *0.38, *P *=* *0.002; *r *=* *0.36, *P *=* *0.004, respectively). Water content and protein: tannin activity also correlated negatively with LMA, but other nonmechanical traits correlated positively (Table 4). Notably, investment in putative chemical defenses (total phenolics and tannin activity) as well as physical defenses (strength, toughness, etc.) increased as mass investment per leaf area increased (Fig. 3).

**Table 4:** Pearson correlations (*r*) of LMA, SI, and leaf nutrients with other measured leaf traits in Chilean matorral species. Values for species measured at multiple sites were averaged (*n *=* *51–63)

	LMA _L_	SI _L_	N_mass__L_	P_mass__L_	N_water__L_	P_water__L_
Nitrogen: phosphorus	0.04	−0.13	0.11	−0.51***	0.42**	−0.32*
Carbon: nitrogen	0.76***	0.88***	−0.96***	−0.81***	0.03	0.04
NDF _L_	0.40**	0.89***	−0.63***	−0.47***	0.01	0.19
ADL _L_	0.45***	0.70***	−0.56***	−0.45***	0.13	0.19
Total phenolics _L_	0.58***	0.56***	−0.66***	−0.59***	0.12	0.10
Protein precipitation _L_	0.50***	0.48***	−0.54***	−0.54***	0.25	0.16
Protein: protein precipitation _L_	−0.65***	−0.64***	0.73***	0.60***	−0.14	−0.18
Water _L_	−0.85***	−0.66***	0.71***	0.73***	−0.66***	−0.53***
Leaf size _L_	0.03	0.29*	−0.21	−0.05	−0.05	0.11
Thickness _L_	0.78***	0.42**	−0.58***	−0.42**	−0.09	−0.02
Tissue density _L_	0.72***	0.56***	−0.58***	−0.61***	0.70***	0.58***
Punch strength _L_	0.75***	0.78***	−0.74***	−0.66**	0.26*	0.24
Specific punch strength _L_	0.21	0.54***	−0.38**	−0.36**	0.27*	0.25
Work to punch _L_	0.80***	0.71***	−0.73***	−0.60***	0.12	0.14
Specific work to punch _L_	0.45***	0.63***	−0.53***	−0.43***	0.14	0.18
*EI*_W__L_	0.88***	0.62***	−0.69***	−0.56***	0.24	0.25

L, log‐transformed for analysis. NDF, neutral detergent fiber; ADL, acid detergent lignin; LMA, leaf dry mass per area; SI, Loveless sclerophylly index; *EI*_W_, flexural stiffness.

Asterisks indicate *P*‐values: *P <* *0.05; **P <* *0.01; ***P <* *0.001.

N_mass_ and P_mass_ were strongly negatively correlated with mechanical traits, and with C:N, NDF, ADL, total phenolics, tannin activity, leaf thickness, and tissue density, and positively with protein: protein precipitation and water content (Table 4). They did not correlate with N_water_ and P_water_ (*P *>* *0.05), and the latter traits correlated with few other traits (Table 3). N:P did not correlate with any mechanical trait (*P *>* *0.05).

**Figure 4:**
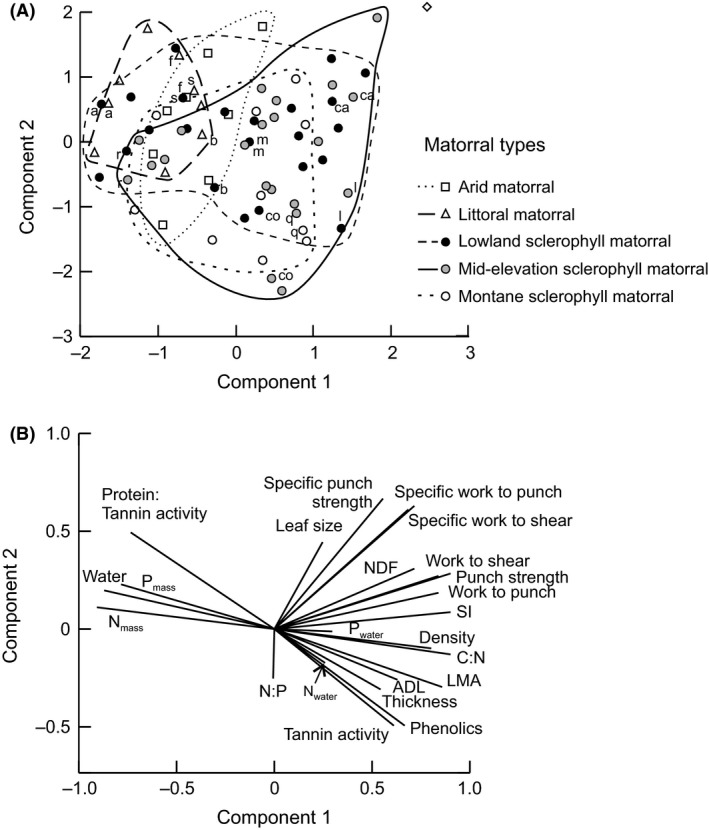
Results of principal components analysis of species across matorral types in Chile, based on measured leaf traits. Traits from tearing and bending tests were not included because they could not be measured across all species. (A) Score plot, with spatial position of species reflecting similarity in leaf traits. Circles, sclerophyll matorral; triangles, littoral matorral; squares, arid matorral; diamond, *Pouteria splendens* at Los Molles. Species sampled from multiple sites were included as individual data points (two could not be included because data were unavailable for some traits): a, *Ageratina glechonophylla*; ca, *Cryptocarya alba;* co, *Colliguaja odorifera*; f, *Flourensia thurifera*; l, *Lithraea caustica*; m, *Maytenus boaria;* r, *Retanilla trinervia*; q, *Quillaja saponaria;* s, *Lepechinia salviae*. Transformation of leaf traits is the same as listed in Table 2. (B) Component loadings plot, showing the relationships of traits with each other and the principal components. NDF, neutral detergent fiber; ADL, acid detergent lignin; LMA, leaf dry mass per area; SI, Loveless sclerophylly index.

These and other correlations are summarized in the PCA plot (Fig. 4A), with 63% of variation among species explained by the first two components. The traits contributing most to the first component (explaining 50% of the variation) were structural strength and toughness (work to punch and to shear), SI, LMA, and C:N (positively), and N_mass_ and water content (negatively) (component loadings ≥0.85) (Fig. 4B). Clustering was consistent with ANOVA trends, but showed more clearly the relationship between the sclerophyll matorral and other forms of matorral (Fig. 4A). In particular, littoral matorral showed little overlap with inland matorral, but overlapped substantially with lowland sclerophyll matorral, which occurred near the coast and also experiences fogs. Second, the three forms of sclerophyll matorral showed considerable trait similarity, but with wider variation in the lowland sclerophyll matorral (Fig. 4A). Arid matorral species had traits intermediate between those of littoral and inland matorral. It was also evident that, overall, leaves investing highly in mass per area were, on average, thicker, denser, stronger, tougher, with more fiber and lignin, more fiber per unit protein, more phenolics, less protein per unit tannin activity, and less N, P, and water per dry mass (Fig. 4B).

Some differences were recorded within species sampled from multiple vegetation types, such as slightly higher water content in populations in littoral matorral than at other sites. However, generally there was little difference between sampled populations, as summarized in the PCA (Fig. 4A).

Soil and climate variables were not intercorrelated (see Table 1 for climate variables tested), except that P_soil_ correlated negatively with AP: ET_0_ (Spearman correlation, *r*_S_ = −0.98, *P *=* *0.005; *n *=* *5 sites) and AMT (annual mean temperature) negatively with ET_0_ (*r*_S_ = −0.98, *P *=* *0.005; *n *=* *5). There was no correlation of LMA, punch traits, or *EI*_W_ with N_soil_ or P_soil_ (*r*_S_ = −0.11 to 0.08, *P *=* *0.32–0.99; *n* species = 61–75). LMA correlated negatively with AMT (*r*_S_ = −0.43, *P *<* *0.001; *n* species = 74) and positively with moisture variables (*r*_S_ = 0.31–0.34, *P* = <0.001–0.008; *n* species = 74) except AP: ET_0_, that is, generally opposite to the direction expected if there was a causal relationship, except for ET_0_. Of the punch variables, only work to punch and punch strength correlated with any climate traits: weakly and negatively with AMT (*r*_S_ = −0.24, *P *=* *0.040; *n* species = 75) for the former and positively with AP (*r*_S_ = 0.23, *n *=* *75: *P *=* *0.047; *n* species = 75) for the latter, again, generally opposite to the direction expected if causal. *EI*_W_ also correlated negatively with AMT (*r*_S_ = −0.36, *P *=* *0.005; *n* species = 61) and positively with PDQ (precipitation of the driest quarter) (*r*_S_ = 0.27, *P *=* *0.037; *n* species = 61), and notably, positively with ET_0_ (*r*_S_ = 0.31, *P *=* *0.016; *n* species = 61)_._ Work to shear, specific work to shear, and PCA Component 1 did not correlate with any environmental variables (*P *>* *0.05).

### Leaf trait relationships with soil and climate across Southern Hemisphere regions

Trends in leaf traits recorded among Chilean species were largely also recorded across the full dataset of the Southern Hemisphere regions, despite deciduous species being absent from the vegetation of both the non‐Chile study areas. LMA was strongly positively correlated (log–log) with most mechanical traits (excluding tearing tests which were not undertaken in Western Australia) across the full dataset across regions (*r *=* *0.41–0.85, *P* = <0.001–0.009). The exception was with specific punch strength (*r *=* *0.29, *P *=* *0.078). Strongest associations were with structural traits (*EI*_W_, followed by strength and work, *r* ^2^ = 0.52–0.72).

**Figure 5:**
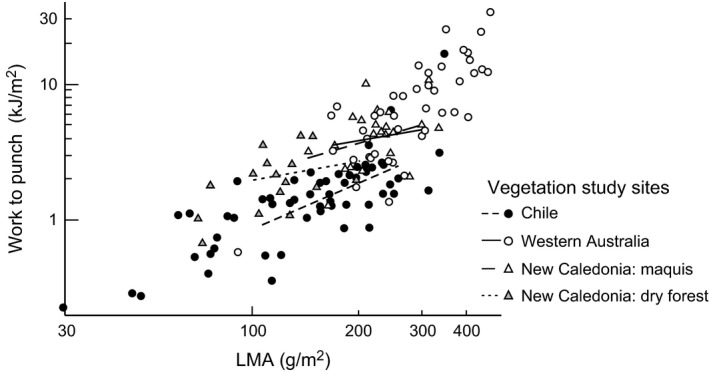
Relationships of work to punch with leaf dry mass per area (LMA) across evergreen shrub and tree species from Chile, New Caledonia, and southwest Western Australia. Species from the littoral matorral at Zapallar are excluded. The line of best fit (OLS regression) is shown for each study region for LMA of 100–300 g m^−2^, the range over which ANCOVA was undertaken.

**Figure 6:**
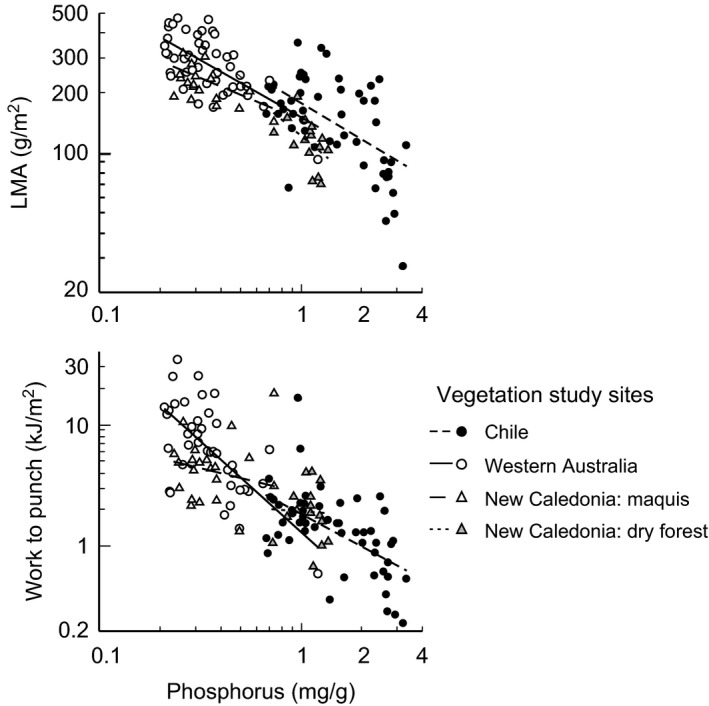
Relationships of leaf dry mass per area and work to punch with foliar P concentration (P_mass_) across evergreen shrub and tree species from Chile, New Caledonia, and southwest Western Australia. Species from the littoral matorral at Zapallar are excluded. The line of best fit (OLS regression) is shown for each study region.

**Figure 7:**
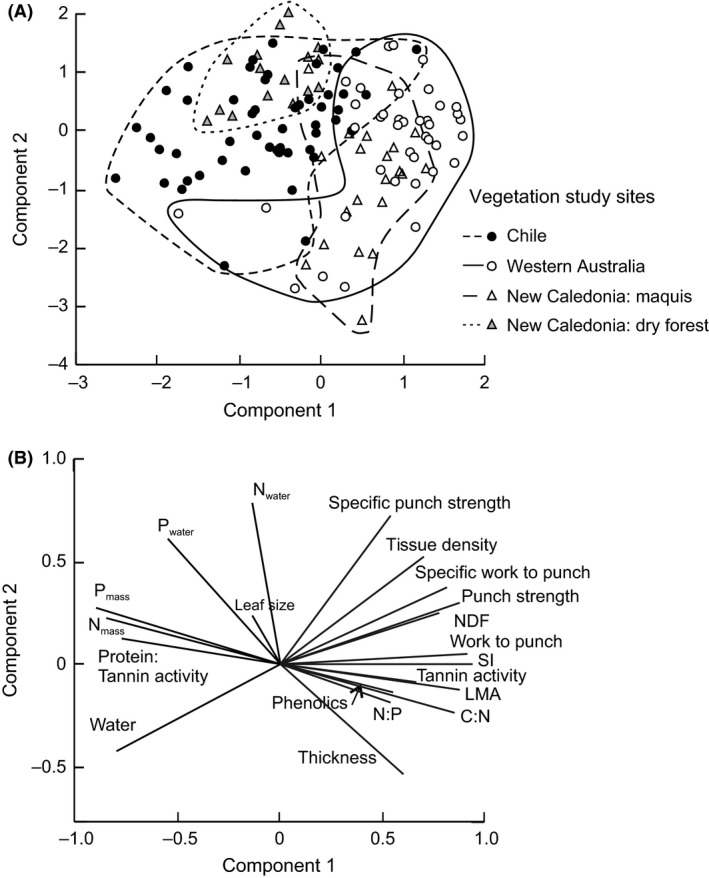
Results of principal components analysis of evergreen shrub and tree species from Chile, New Caledonia, and southwest Western Australia. The same leaf traits were used in this analysis as shown in Figure 4, except that only punch data were used of the mechanical traits, and excluding acid detergent lignin (not measured in earlier studies), and the littoral matorral at Zapallar. (A) Score plot, with spatial position of species reflecting similarity in leaf traits. Average trait values were used for species sampled from multiple sites. Transformation of leaf traits is the same as listed in Table 2. (B) Component loadings plot, showing the relationships of traits with each other and the principal components. NDF, neutral detergent fiber; LMA, leaf dry mass per area; SI, Loveless sclerophylly index.

For evergreen species, the relationship of work to punch with LMA differed significantly among regions (ANCOVA: *F *=* *13.3; *P *<* *0.001), with lower values of work for a given LMA in Chilean matorral than in other regions (*P* = <0.001–0.004) (Fig. 5). Second, across all evergreen species, LMA and all punch variables were strongly negatively correlated with P_mass_ and N_mass_ (log–log relationship) (Table 5, Fig. 6), as in Chilean matorral. Notably, they also correlated negatively, but more weakly, with P_water_, except specific punch strength (Table 5), in contrast to the Chilean matorral. Only specific punch strength correlated with N_water_ across regions, but positively (Table 5). These and other correlations are summarized by the PCA (Fig. 7A). The first component explained 51% of the total variance, with SI, work to punch, P_mass_, LMA, and punch strength contributing most strongly (component loadings >0.85), and N_water_ and specific punch strength contributing most to the second component explaining 14% of the variance (component loadings >0.70) (Fig. 7B). Overall, leaves of evergreen species in West Australian woodland and shrubland and New Caledonian maquis were tougher, stronger, with higher SI and LMA, lower N_mass_ and P_mass_, and lower protein: tannin activity than plants in Chilean matorral and New Caledonian dry forest (Fig. 7A and B).

**Table 5:** Relationships of leaf dry mass per area (LMA) and mechanical traits (punch variables) with nutrient concentration in leaf and soil, and with climate variables, in evergreen species from shrubland and woodland in three regions: southwest Western Australia, New Caledonia, and Chile (data from this paper, excluding the littoral Zapallar site). The data are Pearson *r*‐values (*n *=* *140, across nine soils and seven climates)

	LMA _L_	Strength _L_	Specific strength _L_	Work _L_	Specific work _L_
P_mass L_	−0.74***	−0.64***	−0.27**	−0.77***	−0.63***
P_water__L_	−0.46***	−0.34***	0.03	−0.54***	−0.37***
N_mass L_	−0.73***	−0.60***	−0.26**	−0.63***	−0.48***
N_water__L_	−0.17	0.01	0.29**	−0.13	0.06
P_soil L_	−0.57***	−0.53***	−0.24**	−0.63***	−0.53***
N_soil L_	−0.27**	−0.13	0.08	−0.17*	−0.05
AMT _L_	−0.06	0.14	0.19*	0.16	0.24**
AP _L_	0.07	0.13	0.07	0.18*	0.17
PDQ _L_	0.31***	0.35***	0.20*	0.45***	0.43***
ET_0 L_	0.52***	0.51***	0.27**	0.66***	0.60***
AP: ET_0 L_	−0.22	−0.17	−0.04	−0.23	−0.16

L, log‐transformed for analysis. AMT, annual mean temperature; AP, annual precipitation; PDQ, precipitation of the driest quarter; ET_0_, annual reference evapotranspiration. Climate data were obtained as described for Chile study sites, except for New Caledonia where AMT was estimated by WorldClim v. 1.4 (Hijmans et al. 2005; 30 arc seconds resolution) with DIVA‐GIS v. 7 (Hijmans et al. 2012), and AP from local stations and isohyet maps (Read et al. 2006) (Appendix S3).

Asterisks indicate *P*‐values: *P <* *0.05; **P <* *0.01; ***P <* *0.001.

**Figure 8:**
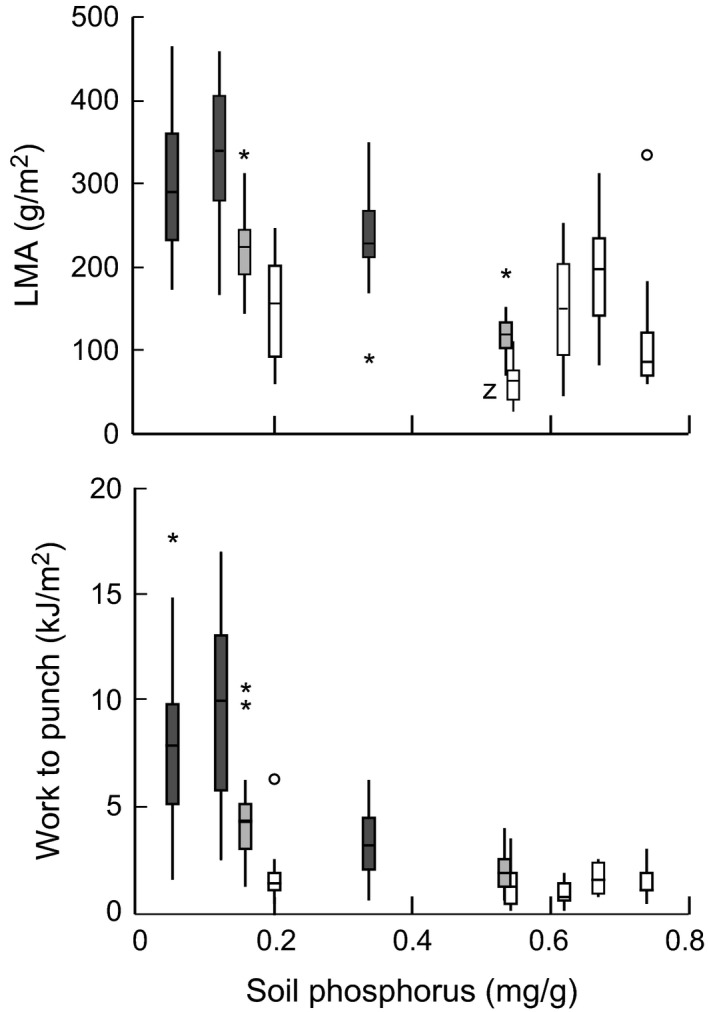
Relationships of leaf dry mass per area and work to punch with total soil P (P_soil_) in evergreen shrub and tree species from Chile, New Caledonia, and southwest Western Australia. Box plots are shown for each vegetation. The littoral matorral at Zapallar is included, indicated by “z”. Chile sites, white; New Caledonia sites (maquis and dry forest), light gray; Western Australia sites (on dolerite and laterite soils and gray sand), dark gray.

Stronger correlations were recorded with environmental variables across regions than across Chilean matorral. LMA and mechanical traits were strongly negatively correlated with P_soil_ and positively with ET_0_ and PDQ (Table 5), the latter in the opposite direction to that expected if causal. When values were averaged for sites, most mechanical traits and LMA correlated strongly and negatively with P_soil_ across regions (*r *=* *−0.86 to −0.68; *P *=* *0.002–0.035) (Fig. 8), and positively with ET_0_ (*r *=* *0.68–0.87; *P *=* *0.003–0.044) (all variables log‐transformed). In addition, the first component of the PCA correlated negatively with P_soil_ (*r *=* *−0.87, *P *=* *0.003) and positively with ET_0_ (*r *=* *0.78, *P *=* *0.021) (environmental variables log‐transformed). Only P_soil_ and ET_0_ made significant independent contributions to variation in punch variables among regions (Table 6), with P_soil_ contributing singly (strength and specific strength) or with ET_0_ (work and specific work) up to 90% of the variation in the dependent variables (Table 6). High but nonsignificant independent contributions were made by P_soil_ and ET_0_ to LMA (Table 6).

**Table 6:** Independent contributions of soil nutrient concentrations and climate variables to mechanical traits (punch variables) and LMA in evergreen species from shrubland and woodland in three regions: southwest Western Australia, New Caledonia, and Chile (data from this paper, excluding the littoral Zapallar site). The data presented are *I*_HP_, the independent contribution of predictor variables to explained variance based on hierarchical partitioning, with *Z*‐scores derived by randomization techniques and statistical significance based on the upper 95 percentile of the normal distribution (*Z *≥* *1.65, shown in bold type). The data used in the analysis were site averages (*n *=* *9 sites). *F‐* and *r* ^*2*^‐values are presented from regression of significant predictor variables; in the case of LMA, the two variables with high but nonsignificant *Z*‐values were included

	LMA_L_		Strength_L_		Specific strength _L_		Work _L_		Specific work _L_	
	*I*_HP_	Z	*I*_HP_	Z	*I*_HP_	Z	*I*_HP_	Z	*I*_HP_	Z
P_soil L_	0.30	1.38	0.43	**2.47**	0.35	**2.00**	0.35	**1.80**	0.33	**1.98**
N_soil L_	0.07	−0.46	0.08	−0.26	0.10	−0.16	0.06	−0.57	0.06	−0.76
AMT _L_	0.11	−0.20	0.03	−0.94	0.17	0.46	0.02	−0.84	0.06	−0.69
AP _L_	0.07	−0.50	0.04	−0.78	0.08	−0.27	0.05	−0.76	0.06	−0.53
PDQ _L_	0.14	0.13	0.13	0.26	0.10	−0.33	0.16	0.34	0.16	0.19
ET_0 L_	0.28	1.45	0.27	1.28	0.19	0.61	0.34	**2.70**	0.32	**2.06**
*F*	7.5*		19.3**		5.9*		27.1**		19.7**	
*r* ^2^	0.72		0.73		0.46		0.90		0.87	

L, log‐transformed for analysis; LMA, leaf dry mass per area; AMT, annual mean temperature; AP, annual precipitation; PDQ, precipitation of the driest quarter; ET_0_, annual reference evapotranspiration.

Asterisks indicate *P*‐values for regressions: *P <* *0.05; **P <* *0.01.

## Discussion

### Variation in matorral leaf form

Leaf form varied considerably among Chilean matorral species, including within types of matorral (see also Rundel 1981). Some species were summer‐deciduous, with LLS of *c*. 3–4 months (e.g., Mooney and Kummerow 1971), but most were evergreen, although with a LLS range of <1 year to *c*. 3 years. A small number of evergreen sclerophylls, such as *Kageneckia angustifolia (*Cavieres et al. 2007), *Quillaja saponaria,* and *Colliguaja odorifera,* show significant leaf loss during summer (Rundel 1981). Differences in rooting depth and physiology among species are likely to be associated with variation in leaf habit (Giliberto and Estay 1978; Montenegro et al. 1979) and may contribute to the wide variability in leaf mechanics recorded within matorral types.

There was particularly strong variation in structural mechanical traits, from leaves that were soft, often short‐lived (including deciduous species), with high nutrient concentrations, to leaves that were strong, stiff, and tough, having, on average, high LMA, C:N, SI, and tissue density, and low N, P, and water concentration. Species with high LMA tended to be tough with high tannin activity and levels of total phenolics. Hence, additive investment in C‐based mechanical and chemical defenses may be cost‐effective on these low‐nutrient soils where plant carbohydrates may be in surplus, as suggested in other sclerophyll vegetation (Read et al. 2009). N limitation was common, but 70% of species had N:P ratios of 10–20, suggesting growth may be limited by factors other than N or P (Güsewell 2004). This differs considerably from sclerophyll vegetation in Australia (Read et al. 2000, 2005) and in maquis in New Caledonia (Read et al. 2006), where N:P ratios are generally high, with low P_mass_, suggesting strong P limitation (Güsewell 2004).

### Variation in sclerophylly among types of matorral

Environmental severity was high in the arid matorral (aridity), the mid‐elevation sclerophyll matorral (aridity and low‐nutrient soils), and the montane sclerophyll matorral (low winter temperatures and aridity). In particular, the sclerophyll matorral vegetation types contained many species with strong, tough, and stiff leaves, both at the structural level and per unit leaf thickness, with high LMA and tissue density, and with notably low levels of protein: tannin activity, and high levels of phenolics, tannin activity, and ADL. Hence, they were potentially well protected against biotic and abiotic stresses. These associated leaf traits form a broad sclerophyll syndrome that has been reported elsewhere, consistent with low soil fertility and/or seasonal dryness (Read et al. 2005, 2006).

However, despite the relatively severe conditions, there was considerable within‐vegetation trait variability, as noted in other studies (Ordoñez et al. 2009). In the arid matorral, this was largely due to a high representation of soft‐leaved deciduous species, as well as strong and tough evergreen species. Similarly, in the sclerophyll matorral, trait variability appeared largely related to the range of LLS among species, particularly in the lowland sclerophyll matorral due to environmental amelioration (moisture, N, and temperature) related to fog (e.g., Weathers et al. 2000). It is expected that if traits were weighted by species abundance, much stronger patterns in leaf traits would be evident among the matorral types. For example, tough‐leaved species such as *Lithraea caustica* (Anacardiaceae) and *Cryptocarya alba* (Lauraceae) dominated the low–mid‐elevation sclerophyll matorral. Nevertheless, it is clear that a range of plant strategies, exemplified by the range of leaf trait combinations observed within sites, are successful in these low‐resource environments, probably reflecting a range of variation in biomass partitioning, physiology, and phenology (Giliberto and Estay 1978; Montenegro et al. 1979; Rundel 1981), as well as within‐site environmental heterogeneity.

The littoral matorral must also experience stress from exposure to coastal winds and salinity. Leaf traits contrasted greatly with those of sclerophyll matorral species, with low values of most mechanical traits, LMA, and tissue density, as well as other traits associated with defense. These differences are probably largely due to physiological and morphological responses to the overriding stress imposed by the saline windy environment, but may in part be indirect effects, related to the apparently shorter LLS of many species in this vegetation.

Environmental complexity (including effects of fog) and low statistical power limited explanation of variation in leaf traits among types of matorral. There was strong negative correlation of LMA, SI, and mechanical traits with P_mass_ and N_mass_ across species. However, this trend is in part due to dilution of foliar nutrients per unit mass by cell wall in scleromorphic species, as suggested by lack of trends in P_water_ and N_water_ (see also Read et al. 2005, 2006), and there were no significant correlations of LMA or mechanical traits with N_soil_ or P_soil_. There was also little correlation with climate variables in a direction likely to indicate causality, although some components of scleromorphy may enhance frost resistance (Larcher 2005) and contribute to negative correlations of LMA, work, and *EI*_W_ with AMT. Nevertheless, the positive correlation of *EI*_W_ and LMA with ET_0_ suggests water availability has a strong influence on variation in leaf structure among these types of matorral.

### Associations of mechanical traits with soil and climate across Southern Hemisphere regions

Summer‐deciduous species were more common in Chilean matorral (see also Lamont 1994) and LLSs of evergreen species were shorter on average than those in some sclerophyll communities in southwest Western Australia, for example, mode of 3 years (J. Read, unpubl. data). Leaves of evergreen Chilean matorral species were on average softer, with lower LMA, higher nutrient content per mass, and less defended than those of southwest Australia or maquis in New Caledonia. LMA and mechanical traits of Chilean matorral species rarely reached the high values seen in species from the latter vegetation types. Soil total P and ET_0_ were the only environmental variables that made a significant independent contribution to variance in mechanical traits of evergreen species among regions, even given potential N limitation in some Chilean species. Total N is not necessarily a good indicator of soil N availability, possibly contributing to its often poor relationships with leaf traits, whereas total P may provide a fairly robust index of P fertility (Ordoñez et al. 2009). Notably, P_water_, an estimate of protoplasmic P concentration, was also negatively correlated with LMA and most mechanical traits across these vegetation types. Interestingly, ET_0_ contributed independently only to work and specific work, rather than to strength and specific strength, suggesting a different anatomical basis of these mechanical traits.

Overall, somewhat similar tradeoffs between leaf traits associated with growth versus resource conservation were suggested across these shrublands and woodlands to those noted in global comparisons of leaf traits across a much wider variety of vegetation types (e.g., Wright et al. 2005; Ordoñez et al. 2009; Onoda et al. 2011). Our study suggests important independent roles of moisture availability (ET_0_) and soil P in variation in leaf texture (LMA and leaf mechanics, the effects differing among mechanical traits) across shrubland and woodland at a broad geographic scale, with the combination of ET_0_ an d soil P explaining 87–90% of variation in work to shear and specific work to shear. Important roles of soil P and moisture availability have been shown recently in photosynthetic and associated leaf traits at a global scale (Maire et al. 2015). Notably, precipitation: ET_0_ (Maire et al. 2015) was not correlated with LMA and mechanical traits in our study, probably due to high variability in rainfall seasonality across the small number of sites.

If sclerophylly is predominantly a consequence of evolved responses to low availability of either nutrients or water, different anatomical traits may contribute to the variation in leaf textures among differing environments, depending on the degree to which each factor is limiting, or has an additive or interactive effect. In contrast, if sclerophylly has evolved predominantly by enhancing protection against damage in suboptimal environments, the suites of anatomical features contributing to texture may be more similar among differing environments, although differing environments may influence the efficiency of various forms of protection. If instead sclerophylly is a variable syndrome of traits, to which multiple factors contribute, identifying the relative contribution of direct v. indirect adaptations v. nonadaptive factors is likely to be difficult. Nevertheless, the simple comparison of work to punch with LMA suggests a difference in the anatomical or material basis of sclerophylly in the Chilean species compared with those from the other Southern Hemisphere regions, and possibly a differing adaptive basis. Studies of leaf anatomy (Kummerow 1973; Read et al. 2000) and tissue‐level mechanical traits (Méndez‐Alonzo et al. 2013) across these regions should assist in addressing these questions.

## Conflict of Interest

None declared.

## Supporting information


**Appendix S1.** Comparisons of leaf traits of evergreen species among matorral vegetation types.

**Appendix S2.** Pearson correlation matrix of mechanical traits from shear, punch, tearing and bending tests across Chilean matorral species.

**Appendix S3.** Environmental data from New Caledonia (NC) and Western Australia (WA) used in meta analysis of leaf traits.

## Species sampled in matorral in central Chile, and their life form and leaf habit. Nomenclature was taken from “The International Plant Names Index” (http://www.ipni.org/) and “The Plant List” (http://www.theplantlist.org/) (accessed 16/12/2015) with additional leaf habit (e, evergreen; d, deciduous; sd, semi‐deciduous) and plant habit information from Hoffmann (1978), Montenegro et al. (1979), and Ginocchio and Montenegro (1992). “Subshrub” is used here to refer to weakly woody shrubs. The final column indicates the mechanical tests undertaken: p, punching; s, shearing; t, tearing; b, bending. *Adesmia microphylla* (Fabaceae) occurred in most matorral sites but was only included at Las Chinchillas because of difficulty conducting mechanical tests on the tiny leaflets.

**Table:**

**Arid matorral: Las Chinchillas**			
*Adesmia microphylla* (Fabaceae)	sd	shrub	p
*Bridgesia incisifolia* (Sapindaceae)	d	shrub	ps
*Cordia decandra* (Boraginaceae)	e	shrub	psb
*Flourensia thurifera* (Asteraceae)	d	shrub	all
*Krameria cistoidea* (Krameriaceae)	e	shrub	p
*Llagunoa glandulosa* (Sapindaceae)	e	shrub	p
*Porlieria chilensis* (Zygophyllaceae)	e	shrub, small tree	p
*Proustia ilicifolia* (Asteraceae)	e	shrub, small tree	all
*Proustia cuneifolia*	d	shrub	psb
*Senna arnottiana* (Caesalpiniaceae)	e	shrub	psb
**Littoral matorral: Zapallar**			
*Ageratina glechonophylla* (Asteraceae)	e	subshrub	all
*Baccharis vernalis* (Asteraceae)	e	shrub	pst
*Bahia ambrosioides* (Asteraceae)	e	subshrub	ps
*Flourensia thurifera*	d	shrub	all
*Fuchsia lycioides* (Onagraceae)	sd	shrub	all
*Haplopappus foliosus* (Asteraceae)	e	subshrub	pst
*Lepechinia salviae* (Lamiaceae)	e	subshrub	all
*Lobelia polyphylla* (Campanulaceae)	e	shrub	all
*Mathewsia foliosa* (Brassicaceae)	e	subshrub	ps
*Senecio sinuatilobus* (Asteraceae)	e	subshrub	all
**Lowland sclerophyll matorral: Cachagua**			
*Adenopeltis serrata* (Euphorbiaceae)	e	shrub	all
*Aextoxicon punctatum* (Aextoxicaceae)	e	tree	all
*Ageratina glechonophylla*	e	subshrub	all
*Aristeguietia salvia* (Asteraceae)	e	shrub	all
*Azara celastrina* (Salicaceae)	e	small tree	all
*Baccharis vernalis* (Asteraceae)	e	shrub	ps
*Beilschmiedia miersii* (Lauraceae)	e	tree	all
*Calceolaria morisii* (Calceolariaceae)	e	subshrub	all
*Citronella mucronata* (Cardiopteridaceae)	e	tree	all
*Colliguaja odorifera* (Euphorbiaceae)	e	shrub	all
*Cryptocarya alba* (Lauraceae)	e	tree	all
*Escallonia revoluta* (Escalloniaceae)	e	small tree	all
*Fuchsia lycioides*	sd	shrub	all
*Lepechinia salviae*	e	subshrub	all
*Lithraea caustica* (Anacardiaceae)	e	tree	all
*Lobelia excelsa* (Campanulaceae)	e	shrub	all
*Maytenus boaria* (Celastraceae)	e	tree	all
*Myrceugenia correifolia* (Myrtaceae)	e	tree	all
*Myrceugenia obtusa*	e	small tree	pst
*Peumus boldus* (Monimiaceae)	e	tree	all
*Retanilla trinervia* (Rhamnaceae)	d	shrub	all
*Ribes punctatum* (Grossulariaceae)	e	shrub	all
*Schinus latifolius* (Anacardiaceae)	e	small tree	all
*Senna candolleana* (Caesalpiniaceae)	e	shrub (small tree)	all
**Mid‐elevation sclerophyll matorral: Río Clarillo**			
*Aristotelia chilensis* (Elaeocarpaceae)	e	small tree	all
*Azara petiolaris* (Salicaceae)	e	small tree	all
*Baccharis* sp.(Asteraceae)	e	shrub	pst
*Berberis chilensis* (Berberidaceae)	e	shrub	all
*Cestrum parqui* (Solanaceae)	e	shrub	all
*Colliguaja odorifera*	e	shrub	all
*Cryptocarya alba*	e	tree	all
*Drimys winteri* (Winteraceae)	e	tree	all
*Escallonia pulverulenta* (Escalloniaceae)	e	small tree	all
*Escallonia illinita*	e	shrub (small tree)	all
*Haplopappus* sp. (Asteraceae)	e	shrub	all
*Lithraea caustica*	e	tree	all
*Luma chequen* (Myrtaceae)	e	small tree	ps
*Maytenus boaria*	e	tree	all
*Persea lingue* (Lauraceae)	e	tree	all
*Podanthus mitiqui* (Asteraceae)	e/sd	shrub	all
*Psoralea glandulosa* (Fabaceae)	e	small tree	all
*Quillaja saponaria* (Quillajaceae)	e	tree	all
*Retanilla trinervia*	d	shrub	all
*Schinus polygama* (Anacardiaceae)	e	shrub	all
*Teucrium bicolor* (Lamiaceae)	sd	shrub	all
**Montane sclerophyll matorral: Yerba Loca**			
*Colliguaja integerrima* (Euphorbiaceae)	e	shrub	all
*Escallonia illinita*	e	shrub	all
*Guindilia trinervis* (Sapindaceae)	e	shrub	all
*Haplopappus* sp.(Asteraceae)	e	shrub	all
*Kageneckia angustifolia* (Rosaceae)	e	small tree	all
*Kageneckia oblonga* (Rosaceae)	e	tree	all
*Quillaja saponaria*	e	tree	all
*Schinus montanus* (Anacardiaceae)	e	shrub, small tree	all
*Solanum ligustrinum* (Solanaceae)	e	shrub	all
*Trevoa quinquenervia* (Rhamnaceae)	d	shrub, small tree	pst
**Los Molles**			
*Pouteria splendens* (Sapotaceae)^a^	e	shrub	all

a Sampled in matorral at Los Molles (*c*. 32° 13′ S, 71° 31′ W, 70 m asl) and only included in trait correlation analyses.
