# Supplementary material for: Leaf traits in Chilean matorral: sclerophylly within, among, and beyond matorral, and its environmental determinants
Source: Ecol Evol. 2016 Feb 3;6(5):1430–46. doi: 10.1002/ece3.1970 (PMC4739351; doi:10.1002/ece3.1970)
Supplement: Supplementary file 1 — Appendix S1. Comparisons of leaf traits of evergreen species among matorral vegetation types. [file ECE3-6-1430-s001.docx]

**SUPPORTING INFORMATION**

**Article title: Leaf traits in Chilean matorral: sclerophylly within, among and beyond matorral, and its environmental determinants**

Authors: Jennifer Read, Gordon Sanson, María Fernanda Pérez Trautmann

**Appendix S1** Comparisons of leaf traits of evergreen species among matorral vegetation types. The data are means of species’ means, with standard errors, and are given only for traits in which statistical conclusions differ from those given in Table 2. The results of one-factor ANOVA are given, with *post-hoc* Tukey’s tests when significant differences are detected (shared alphabet letters indicate no significant difference among sites). Las Chinchillas was not included in tear tests due to the small number of evergreen species that could be tested, given small leaf size. L, log-transformed for analysis.

Trait arid matorral littoral matorral lowland sclerophyll mid-elevation montane sclerophyll *F* *P*

matorral sclerophyll matorral matorral

P_mass_ (mg g^-1^) _L_ 1.74 ± 0.25^ab^ 3.21 ± 0.51^b^ 1.78 ± 0.20^a^ 1.31 ± 0.15^a^ 1.80 ± 0.25^ab^ **5.3 0.001**

P_water_ (mg g ^-1^ water) _L_ 0.97 ± 0.18^ab^ 0.58 ± 0.10^b^ 1.00 ± 0.10^a^ 0.99 ± 0.06^a^ 1.48 ± 0.24^a^ **6.5 < 0.001**

Nitrogen : phosphorus 14.9 ± 3.0^a^ 8.2 ± 0.8^b^ 10.5 ± 0.7^b^ 12.8± 0.6^a^ 11.9± 1.58^ab^ **5.1 0.001**

Carbon : nitrogen 22.7 ± 4.1^a^ 20.5 ± 2.4^a^ 31.6 ± 3.2^ab^ 36.2 ± 3.0^b^ 29.5 ± 2.9^ab^ **3.3 0.016**

Total phenolics (g GAE 100 g^-1^)  _L_ 3.4 ± 1.3^ab^ 1.9 ± 0.3^a^ 4.5 ± 0.6^ab^ 4.9 ± 0.6^b^ 4.9 ± 1.1^ab^ **2.9 0.030**

Succulence (g m ^-2^) 254 ± 52^ab^ 350 ± 38^b^ 199 ± 11^a^ 204 ± 14^a^ 248 ± 20^a^ **7.5 < 0.001**

Leaf size (mm^2^) _L_ 231 ± 93^a^ 748 ± 236^b^ 1239 ± 212^b^ 1036 ± 210^b^ 421 ± 102^ab^ **6.2 <0.001**

Tissue density (mg mm^-3^) _L_ 0.372 ± 0.038^a^ 0.199 ± 0.021^b^ 0.435 ± 0.024^a^ 0.484 ± 0.022^a^ 0.485 ± 0.031^a^ **13.6 < 0.001**

Punch strength (MN m^-2^) _L_ 5.19 ± 0.71^ab^ 2.96 ± 0.44^a^ 6.23± 0.77^b^ 7.08 ± 0.98^b^ 5.70 ± 0.64^ab^ **3.4 0.015**
