# Supplementary material for: Leaf traits in Chilean matorral: sclerophylly within, among, and beyond matorral, and its environmental determinants
Source: Ecol Evol. 2016 Feb 3;6(5):1430–46. doi: 10.1002/ece3.1970 (PMC4739351; doi:10.1002/ece3.1970)
Supplement: Supplementary file 2 — Appendix S2. Pearson correlation matrix of mechanical traits from shear, punch, tearing and bending tests across Chilean matorral species. [file ECE3-6-1430-s002.docx]

**SUPPORTING INFORMATION**

**Article title: Leaf traits in Chilean matorral: sclerophylly within, among and beyond matorral, and its environmental determinants**

Authors: Jennifer Read, Gordon Sanson, María Fernanda Pérez Trautmann

**Appendix S2** Pearson correlation matrix of mechanical traits from shear, punch, tear and bending tests across Chilean matorral species. Only those species for which all tests could be conducted are included (*n* = 49). Values are averaged for species collected at multiple locations. *P* ≤ 0.001 except where indicated; *, *P* <0.05; ns, not significant.

Work to shear SWS PS SPS WP SWP TS WT SWT E

Specific work to shear (SWS) 0.872

Punch strength (PS) 0.918 0.769

Specific punch strength (SPS) 0.658 0.812 0.770

Work to punch (WP) 0.943 0.730 0.953 0.615

Specific work to punch (SWP) 0.862 0.876 0.876 0.877 0.863

Tear strength (TS) 0.793 0.808 0.791 0.751 0.705 0.754

Work to tear (WT) 0.830 0.670 0.789 0.532 0.826 0.732 0.806

Specific work to tear (SWT) 0.613 0.683 0.584 0.650 0.539 0.673 0.811 0.855

*E* 0.679 0.678 0.651 0.550 0.577 0.552 0.782 0.549 0.500

*EI*_W_ 0.808 0.478 0.739 0.222^ns^ 0.832 0.518 0.570 0.705 0.304* 0.641
