# Supplementary material for: Leaf traits in Chilean matorral: sclerophylly within, among, and beyond matorral, and its environmental determinants
Source: Ecol Evol. 2016 Feb 3;6(5):1430–46. doi: 10.1002/ece3.1970 (PMC4739351; doi:10.1002/ece3.1970)
Supplement: Supplementary file 3 — Appendix S3. Environmental data from New Caledonia (NC) and Western Australia (WA) used in meta analysis of leaf traits. [file ECE3-6-1430-s003.docx]

**SUPPORTING INFORMATION**

**Article title: Leaf traits in Chilean matorral: sclerophylly within, among and beyond matorral, and its environmental determinants**

Authors: Jennifer Read, Gordon Sanson, María Fernanda Pérez Trautmann

**Appendix S3** Environmental data from New Caledonia (NC) and Western Australia (WA) used in meta analysis of leaf traits. Annual mean temperature (AMT) and annual precipitation (AP) were obtained for WA sites from local meteorological stations. For New Caledonia, AMT was estimated by WorldClim v. 1.4 (Hijmans *et al*., 2005; 30 arc-seconds resolution) with DIVA-GIS v. 7 (Hijmans *et al.,* 2012), and AP from local stations and isohyet maps (Read *et al.* 2006). For both regions, monthly precipitation was estimated by WorldClim v. 1.4 with DIVA-GIS v. 7. Penman-Monteith reference evapotranspiration rate (ET_0_) was estimated by the 10-arcmin IWMI World Water Climate Atlas (<http://www.iwmi.cgiar.org/>). PDQ, precipitation of the driest quarter. Soil N and P were taken from Read *et al.* (2005, 2006).

**Vegetation, region AMT AP PDQ ET_0_ AP/ET_0_ soil N soil P**

(°C) (mm) (mm) (mm) (mm) (mg g^-1^) (mg g^-1^)

Maquis, NC 22.6 1820 170 1374 1.32 0.8 0.16

Dry forest, NC 22.9 945 138 1441 0.66 4.9 0.53

Woodland, WA 16.0 446 37 1570 0.28 2.2 0.33

Shrubland (laterite), WA 16.0 446 37 1570 0.28 1.4 0.12

Shrubland (sand), WA 16.0 446 37 1570 0.28 0.1 0.05
